# A chromosome-level genome assembly of a free-living white-crowned sparrow (*Zonotrichia leucophrys gambelii*)

**DOI:** 10.1038/s41597-024-02929-6

**Published:** 2024-01-18

**Authors:** Zhou Wu, Katarzyna Miedzinska, Jesse S. Krause, Jonathan H. Pérez, John C. Wingfield, Simone L. Meddle, Jacqueline Smith

**Affiliations:** 1 The Roslin Institute and Royal (Dick) School of Veterinary Studies R(D)SVS, The University of Edinburgh, Easter Bush, Midlothian, EH25 9RG UK; 2 Department of Neurobiology, Physiology, and Behavior, University of California, Davis, CA 95616 USA; 3 Department of Biology, University of Nevada Reno, Reno, NV 89557 USA; 4 Department of Biology, University of South Alabama, Mobile, AL 36688 USA

**Keywords:** Genome, Ecological genetics

## Abstract

The white-crowned sparrow, *Zonotrichia leucophrys*, is a passerine bird with a wide distribution and it is extensively adapted to environmental changes. It has historically acted as a model species in studies on avian ecology, physiology and behaviour. Here, we present a high-quality chromosome-level genome of *Zonotrichia leucophrys* using PacBio and OmniC sequencing data. Gene models were constructed by combining RNA-seq and Iso-seq data from liver, hypothalamus, and ovary. In total a 1,123,996,003 bp genome was generated, including 31 chromosomes assembled in complete scaffolds along with other, unplaced scaffolds. This high-quality genome assembly offers an important genomic resource for the research community using the white-crowned sparrow as a model for understanding avian genome biology and development, and provides a genomic basis for future studies, both fundamental and applied.

## Background & Summary

The white-crowned sparrow (WCS; *Zonotrichia leucophrys*) is a small passerine bird that is commonly found in North America and has been historically studied to provide understanding of the biology and ecology in wild, free-living birds. Genomic information from common wild-living birds can provide valuable resources for bioscience^1^. There are five recognized sub-species of white-crowned sparrow (*Zonotrichia leucophrys pugetensis*, *gambelii*, *nuttalli*, *oriantha*, and *leucophrys*) with variation in geographic distribution, appearance and migratory behaviour. White-crowned sparrows offer great opportunities to understand the evolution of subspecies through hybridization and introgression that is characterized by the genomic landscape. As a model species for understanding divergence of behavioural and physiological process, genetic methodologies and approaches have been commonly employed to study the underlying mechanisms using genetic markers on mitochondria or across the whole genome^2^. However, to date, a good quality genome assembly for the white-crowned sparrow has not been available. Previous studies investigating the genetics of *Zonotrichia* species often utilize nucleotide polymorphisms in representative segments of the genome, such as microsatellite markers, genotyping-by-sequencing (GBS), SNP arrays developed for closely-related species, and other restriction site-associated DNA sequencing (RADseq) approaches^2–6^. As a high-quality reference assembly was not available for past genetic studies on white-crowned sparrows, assemblies of other bird species were commonly used as a reference, e.g. genomes of the white-throated sparrow (*Zonotrichia albicollis*), zebra finch (*Taeniopygia guttata*), canary (*Serinus canaria*) or chicken (*Gallus gallus*)^7–10^. The compatibility of these types of studies could be greatly improved by using a specific reference genome assembly and gene models of the white-crowned sparrow.

To this end, we present a high-quality chromosome level genome assembly for the white-crowned sparrow using the subspecies *Zonotrichia leucophrys gambelli*. Previous studies suggested that the *Zonotrichia leucophrys* karyotype is 2n = 82^11–13^. This comprises several pairs of micro-chromosomes, characterized by small size and higher gene density, which is a feature of bird karyotypes^13^. We combined long-read sequencing (PacBio) and information on DNA compartment proximity (Omni-C) to generate a genome of 1,123,996,003 bp, including 3,792 scaffolds with a scaffold N50 of 72 Mb. We assembled 31 relatively complete chromosomes, representing all macro-chromosomes (including the Z sex chromosome), most of the intermediate chromosomes and a good number of micro-chromosomes.

## Methods

### Sample collection

Samples were collected from two wild, free-living female Gambel’s white-crowned sparrows (*Zonotrichia leucophrys gambelli*) captured on breeding grounds in the vicinity of Toolik Lake Research Station on the North Slope of Alaska (N 68° 45′, W149° 52′) on 28^th^ May 2016 (for DNA extraction) and 20^th^ July 2016 (for RNA extraction). There were no severe weather perturbations (e.g., snowstorm) observed on the days of collection. Following capture with a mist net, a blood sample was collected within three minutes of capture by venipuncture of the alar vein with a 26-gauge needle and transferred into heparinized glass microcapillary tubes (VWR: 15401-56). The birds were quickly sedated with isoflurane and euthanized within three minutes. Following euthanasia, the left pectoralis muscle, brain, liver and ovary were dissected, flash frozen on dry ice, wrapped individually in aluminium foil into labelled plastic bags and kept frozen on dry ice until they were stored in a −80 °C freezer upon returning to the laboratory.

For DNA extraction, a frozen sample of pectoralis muscle from one individual was sent on dry ice to Dovetail Genomics (California, USA). The RNA samples from the other individual were later shipped on dry ice to the Roslin Institute, University of Edinburgh, UK, where they were stored at −80 °C. Approximately 100 mg of liver and ovarian tissue was homogenized for RNA extraction and for the hypothalamus we used 150 mg of tissue.

The work was approved by the University of California, Davis, USA Institutional Animal Care and Use Committee (AICUC) under protocol 19758, United States Fish and Wildlife Service - Federal MB90026B-0 and The Animal Welfare and Ethical Review Body at the Roslin Institute, The University of Edinburgh, UK.

### Genome sequencing

Pectoralis muscle was used to obtain high molecular weight DNA (50 to100 Kb), which was subsequently used for PacBio library preparation after satisfactory quality control. The library preparation, sequencing and scaffolding were carried out by Dovetail Genomics (California, USA) according to their standard genome assembly pipeline (https://dovetailgenomics.com/). In short, the PacBio SMRTbell library was constructed using SMRTbell Express Template Prep Kit 2.0 (PacBio, Menlo Park, CA, USA). Sequencing of the genome was performed with PacBio Sequel II 8 M SMRT cells, yielding 273.6 Gb data. Sequences were then assembled into scaffolds by using Wtdbg2^14^, followed by contamination detection and duplicated haplotig purging using Blobtools (v2.9)^15^ and purge_dups (v1.1.2)^16^ respectively.

A proximity ligation library was generated by the Omni-C technique^17^, followed by sequencing on an Illumina HiSeqX platform. Chromatin was fixed in place in the nucleus with formaldehyde before extraction (for technical note, see https://dovetailgenomics.com/wp-content/uploads/2021/09/Omni-C-Tech-Note.pdf). Fixed chromatin was digested with DNAse I, fragmented chromatin ends were repaired and biotinylated to adapters followed by proximity ligation. Crosslinks were then reversed, the DNA purified and the biotin subsequently removed. The DNA library was prepared and sequenced to produce 2 × 150 bp paired-end reads at a coverage of around 30X. The Omni-C technology uses a sequence-independent endonuclease which provides even, unbiased genome coverage. The HiRise pipeline was employed for further scaffolding of the *de novo* assembly^18^. The genome assembly and Omni-C sequences were used as input for the HiRise pipeline, mainly to determine genomic distance between proximity ligation reads to identify the joins and mis-joins within the scaffolds. The interaction matrix was corrected (–filterThreshold −2.5 3) and visualized by HiCExplorer (V3.7.2)^19^ (supplementary file 1 Figure S1). In addition, we used short-read sequences from a WCS individual (the same one used in RNA-sequencing) to perform genome polishing, using POLCA^20^ and pilon (v1.24)^21^ with default parameters.

### RNA-seq sample preparation and sequencing

In order to generate a gene model for the white-crowned sparrow genome, we used three RNA-sequencing datasets of the brain (specifically the hypothalamus), liver, and ovary from an individual independently. To isolate RNA for RNA-sequencing, RNA samples were homogenized in TRIzol reagent (Invitrogen) and the Direct-zol RNA Miniprep kit (Zymo Research USA) protocol was followed for RNA extraction. After elution of the total RNA in RNAse-free water, we ensured a minimum of 500 ng RNA with a concentration of >12.5 ng/µL for library preparation. The library construction involved PolyA selection and subsequent sequencing on the BGI DNBSEQ platform, generating 150 bp paired-end reads and around 30 million sequences per read. The BGI DNBSEQ is comparable to the Illumina platform that allows high-quality short-read second generation sequencing^22,23^. The reads were mapped to the genome using STAR (version 2.7.8a)^24^ with default options. The RNA-seq data were used to assist the gene model annotation and the mapping rate was also used to validate the completeness of the assembly.

### Iso-seq library preparation and sequencing

The same 3 RNA samples (hypothalamus, liver and ovary) were further prepared for long-read isoform sequencing (Iso-seq). Previous studies have shown the power of Iso-seq for discovering novel and full-length transcripts and how it can complement RNA-seq data in the annotation of other species, e.g., in chicken^25^. Using the two complementary techniques will provide the advantages of each technique and help us generate a better representative annotation profile. We implemented quality control (QC) using three available methods: NanoDrop spectrophotometer (Thermo Fisher, USA), Qubit 3 fluorometer (Invitrogen, US), and the Tapestation 4200 system (Agilent, US). The starting concentration of the samples were 324 ng/ul, 46 ng/ul and 44 ng/ul, respectively, with RIN > 8. To ensure the quantity of RNA for Iso-seq, libraries were prepared in three technical replicates for ovary and in four technical replicates for liver and hypothalamus. The amount of RNA used for a single reaction was: 0.5 µg for ovary and liver, and 2 µg for hypothalamus. The full-length cDNA was produced using the Teloprime full-length cDNA amplification kit (v1) from Lexogen (cat. No 013.24) according to manufacturer’s protocols. To determine the Optimal Endpoint PCR (OEP) cycle, a qPCR assay was performed on an aliquot of the full-length double-stranded cDNA using a Light Cycler 480 SW 1.5 machine, and the OEP was determined at 20 cycles corresponding to 80% of the maximum fluorescence value (plateau phase) on the amplification curve. Subsequently, the libraries were purified on columns provided by the manufacturer and the technical replicates were then pooled and subjected to QC. The average concentration of each library was 40 ng/µl. The size distribution, as confirmed by the D5000 screen tape on the Tapestation, ranged from 600 to 2500 bp with a significant peak observed around 1500 bp. Full-length cDNA were then used for PacBio SMRT sequencing on the Sequel system (version 2.1). In total, PacBio Iso-seq generated 112 GB data, including 47,186,447 subreads with an average length of 1,389 bp. circular consensus sequences (CCSs) were then created, which subsequently produced 12,219 full-length non-chimeric (FLNC) reads with poly-A tail.

### Genome quality assessment and chromosome assignment

**Fig. 1:**
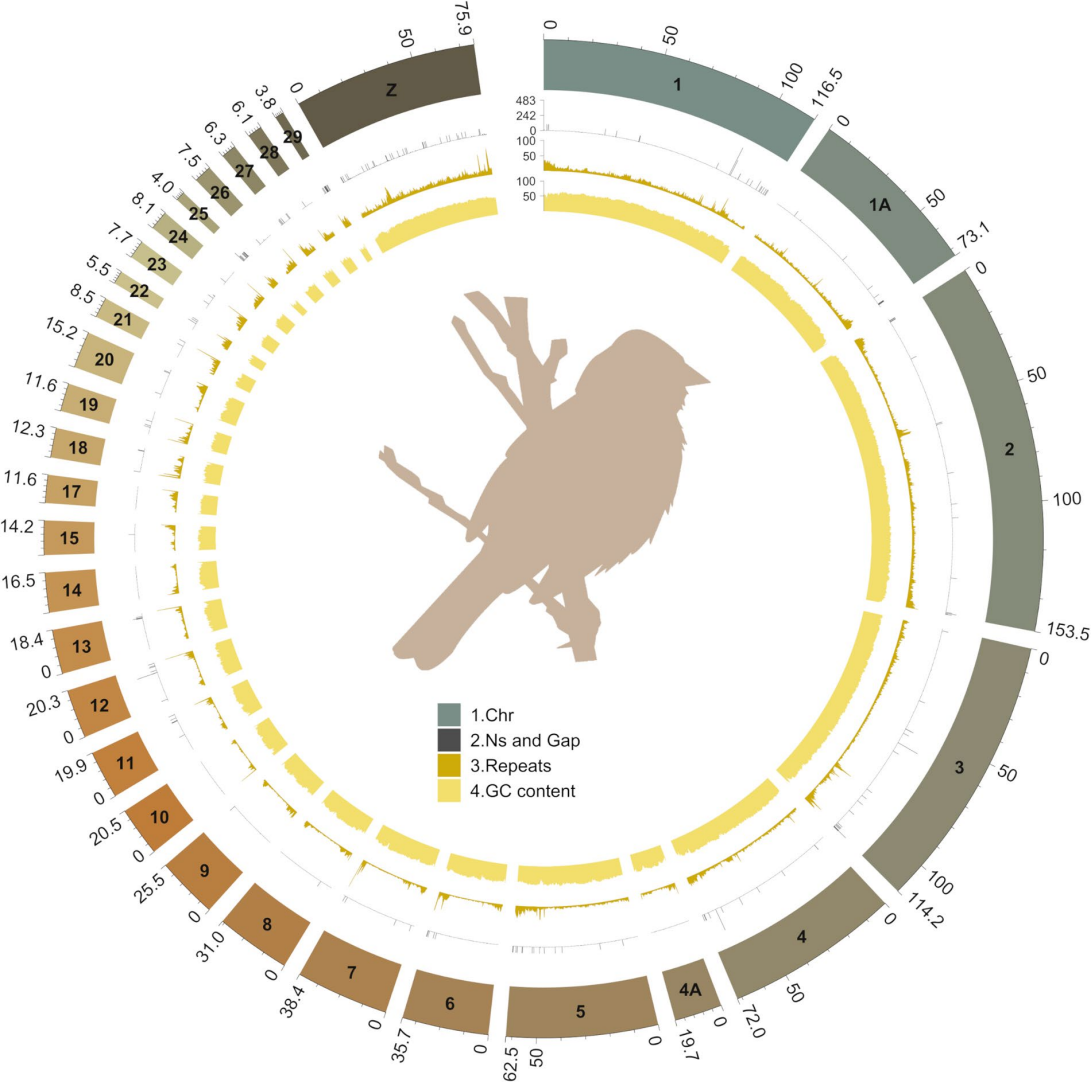
Overview of the genome assembly of the white-crowned sparrow (*Zonotrichia leucophrys gambelii*). The size of chromosomes is displayed in Mb, the Ns and Gaps are in bp, while repeats and GC content are presented as percentages (window size 200k). The bird silhouette image was downloaded from https://www.phylopic.org/ (provided 2017 Aug 29, by Matt Wilkins) under the Creative Commons (CC0) 1.0 Universal Public Domain Dedication License.

**Fig. 2:**
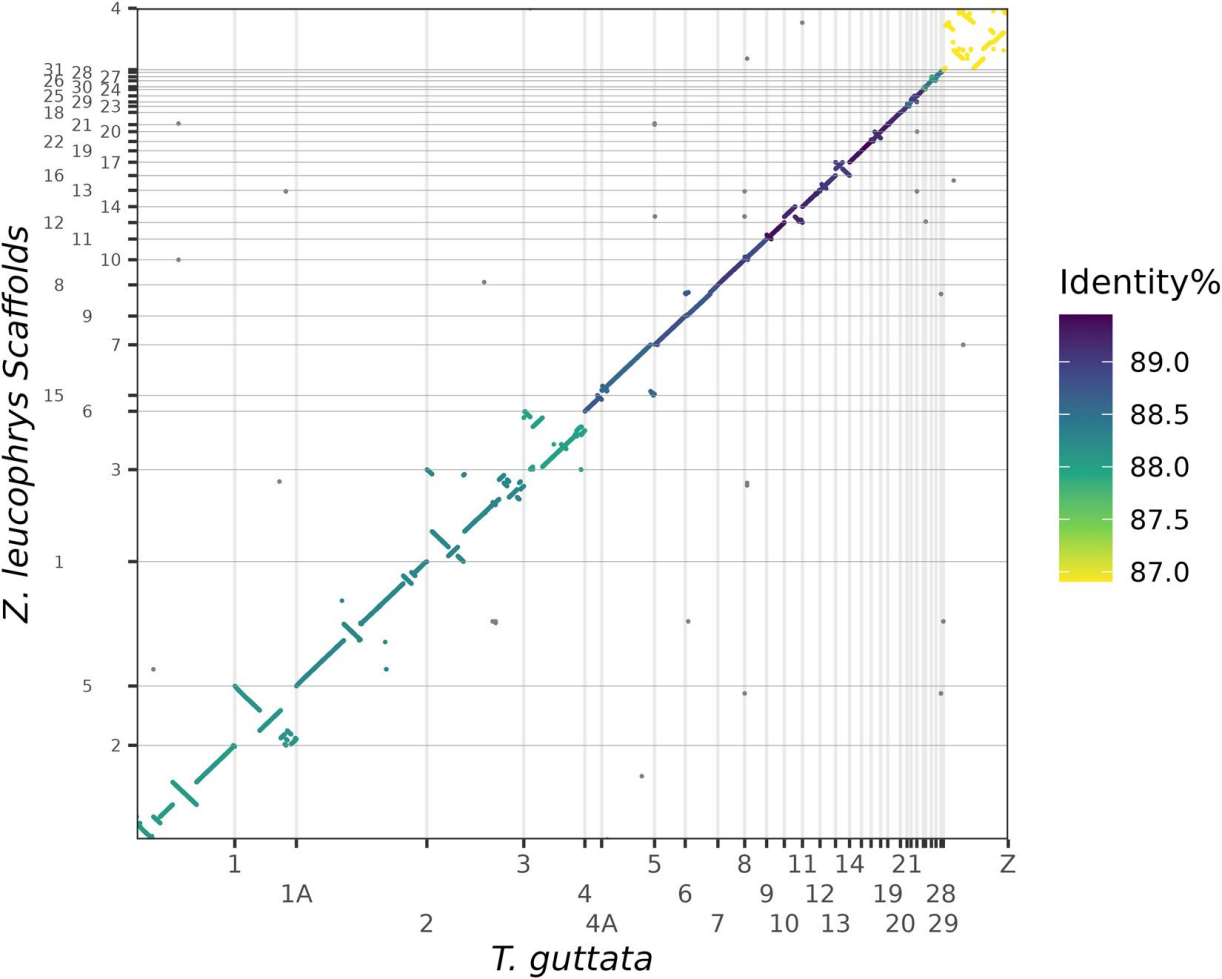
Whole-genome alignment between assemblies of the white-crowned sparrow (*Zonotrichia leucophrys gambelii*) and zebra finch (*Taeniopygia guttata*; version: bTaeGut1.4.pri). The y-axis displays the representative scaffolds of the white-crowned sparrow genome.

**Fig. 3:**
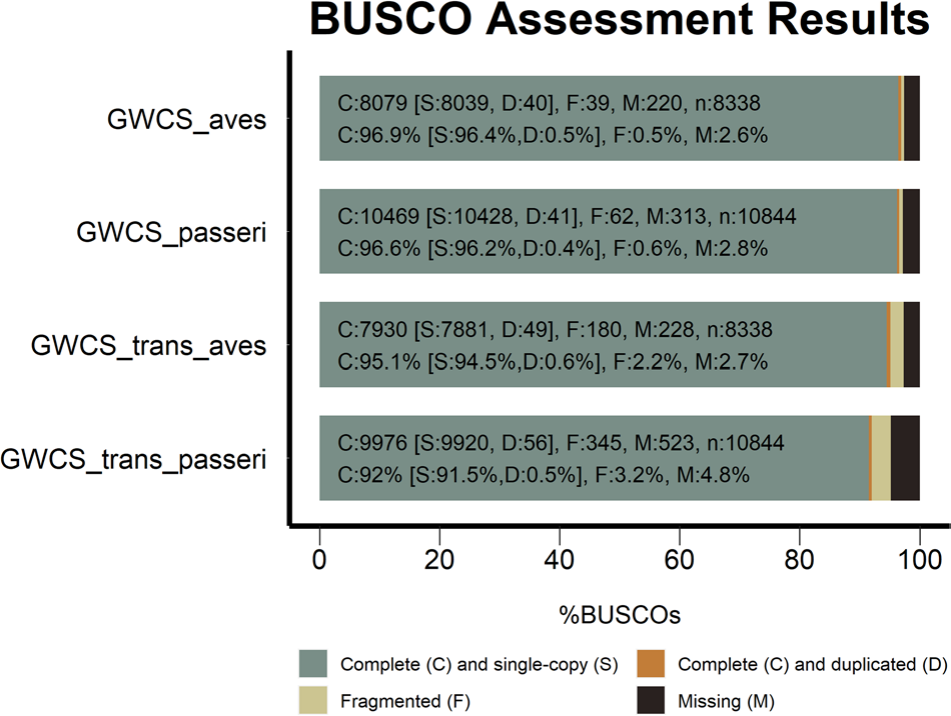
Assessment of Benchmarking Universal Single-Copy Orthologs (BUSCOs) of the white-crowned sparrow (*Zonotrichia leucophrys gambelii*) genome and transcriptome using *aves* and *Passeriformes* (abbreviated as passeri) (odb10) databases.

Thirty-one relatively complete chromosomes have been assembled, including all macro-chromosomes, intermediate chromosomes and most of the micro-chromosomes, representing, 1, 1 A, 2–4, 4 A, 5–15, 17–29, Z (Fig. 1). In total, the size of the Gambel’s white-crowned sparrow genome is 1,123,996,003 bp, including 3,792 scaffolds and 4,117 contigs (Table 1). Chromosome assignment was based on the zebra finch genome assembly (bTaeGut1.4.pri) (Fig. 2). In case of future amendment, the corresponding scaffold assignment is presented in Table 2. In addition, some scaffolds showed shorter alignment to the zebra finch genome. Although we do not have the full confidence to assign them as complete chromosomes, they can tentatively be assumed to represent the chromosomes with complex sequence structure, such as micro-chromosomes 30, 31, 32, 35 and W. These results are separately represented in supplementary file 1 (Figure S2). The prospective chromosomes were visualized by a circos plot using the circlize (v0.4.15)^26^ package in R with annotation of genome characteristics, including Ns and gaps, repeat distribution, and GC content. Completeness of the assembly was assessed with Benchmarking Universal Single-Copy Orthologs (BUSCO) for both the assembled genome sequences and the annotated transcriptome (Fig. 3). The genome has an overall BUSCO score of 96.9% when compared with a total ‘aves’ (odb10) background, with 0.5% duplication, suggesting good completeness and contiguity of the assembly.

**Table 1:** Assessment of the white-crowned sparrow genome assembly.

Assembly features	Gambels_ncbi_update
Counts of scaffold sequences	3,792
Length of scaffold sequences	1,123,996,003
Largest scaffold name	Scaffold_1_153547327
Largest scaffold length	153,547,327
Scaffold N50	71,969,017
Counts of N50	6
Scaffold N90	6,309,133
Counts of N90	27
GC content (%)	42.80%
N Length	26,361
N content (%)	0.002%
Counts of contigs	4,117
Maximum length of contigs	40,609,704
contig N50	14,729,340
Counts of contig N50	25
contig N90	546,537
Counts of contig N90	179

**Table 2:** Chromosome assignment for the white-crowned sparrow assembly.

Scaffold name	Chromosome
Scaffold_2_116484495	1
Scaffold_5_73051372	1 A
Scaffold_1_153547327	2
Scaffold_3_114162194	3
Scaffold_6_71969017	4
Scaffold_15_19713544	4 A
Scaffold_7_62472784	5
Scaffold_9_35708988	6
Scaffold_8_38401667	7
Scaffold_10_31016323	8
Scaffold_11_25524209	9
Scaffold_12_20527583	10
Scaffold_14_19948824	11
Scaffold_13_20270949	12
Scaffold_16_18355265	13
Scaffold_17_16474596	14
Scaffold_19_14189122	15
Scaffold_22_11597714	17
Scaffold_20_12261182	18
Scaffold_21_11615082	19
Scaffold_18_15211132	20
Scaffold_23_8480127	21
Scaffold_29_5518869	22
Scaffold_25_7671743	23
Scaffold_24_8071077	24
Scaffold_30_4037257	25
Scaffold_26_7504969	26
Scaffold_27_6309133	27
Scaffold_28_6126336	28
Scaffold_31_3761913	29
Scaffold_4_75875312	Z

**Fig. 4:**
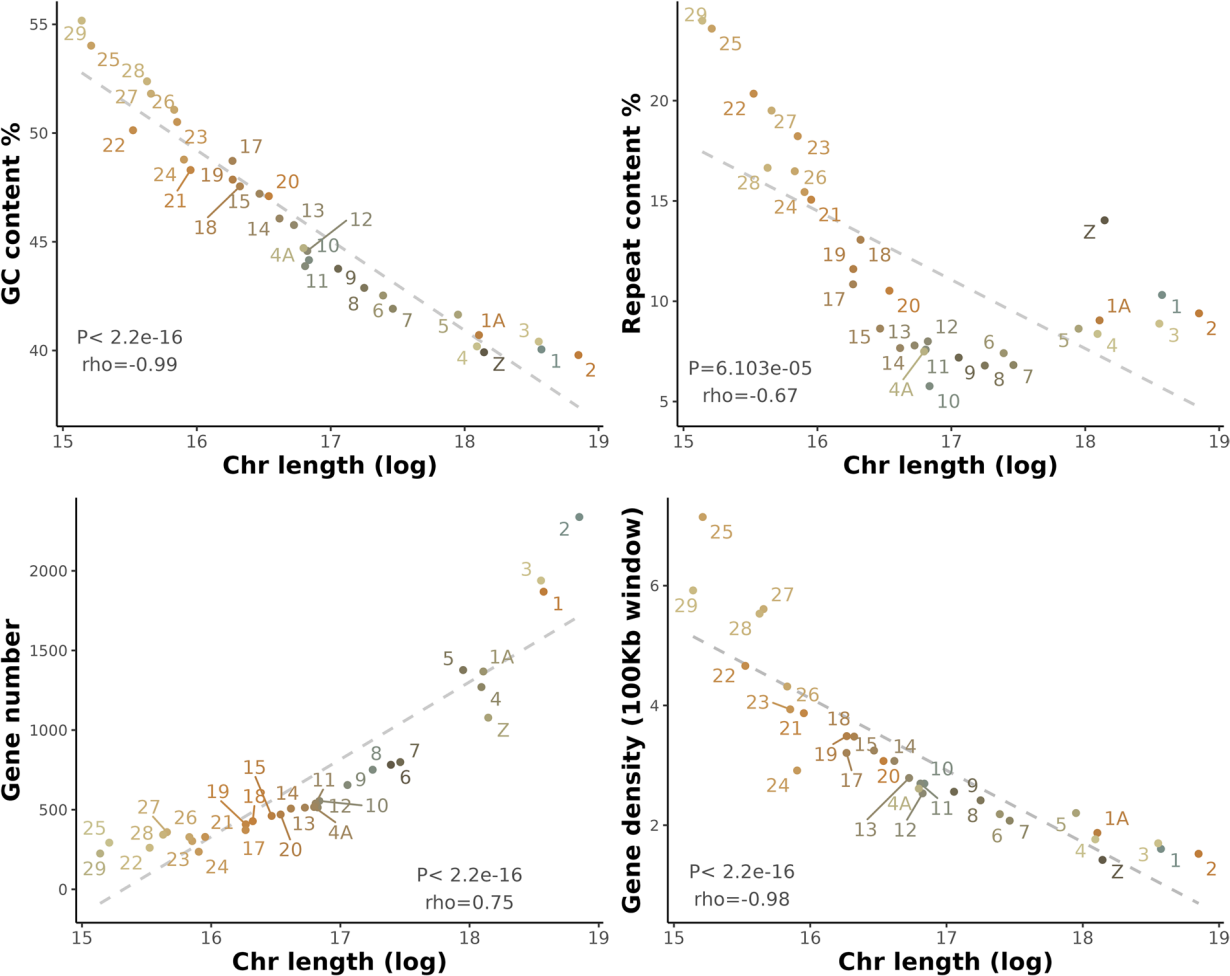
Correlation between chromosome size (shown in x-axis) and GC content, repeat elements, number of genes and gene density (shown in y-axis) of the white-crowned sparrow (*Zonotrichia leucophrys gambelii*) genome. The chromosome size is log transformed and the P value was calculated by Spearman’s test.

The assembly was evaluated by computing quality statistics and detecting repeat elements in the final assembly. First, basic features for the assembly were calculated (e.g., N50, N90, GC content etc.) using available scripts (https://github.com/WenchaoLin/assemblyStatics) (Table 1). The genome assembly shows good contiguity and completeness, with the scaffold N50 being 71.97 Mb, a contig N50 of 14.73 Mb and GC content of 42.80%. In particular, 26,361 bp of Ns are seen in the assembly, making up 0.002% of the total sequence. As for repeat sequences, RepeatModeler (v2.0.2)^27^ was used with the -LTRStruct parameter to firstly build the repeat models (such as transposable element families) and then repeat sequences were annotated and masked in place using RepeatMasker (v4.1.2)^28^ (Table 3). In total, 14.97% of sequences were identified as repeats and soft-masked in the final output. The GC content and repeat content for each chromosome show significantly negative correlation with chromosome size (Fig. 4). This is particularly pronounced in micro-chromosomes, where GC and repeat content are relatively high. Overall, our assembly for the white-crowned sparrow is comparable to previously published genome assemblies of passerine birds in closely-related families (i.e., *Passerellidae* and *Emberizidae*), regarding the genome size (ranging 1.03–1.11 Gb), GC content (41.52–42.75%), repeat content (8.4%–12.19%) and BUSCO score (e.g., complete *aves* BUSCO ranging 91–96.2%)^29,30^.

**Table 3:** Repeat elements identified in the assembly.

Repeats	Count	Length (bp)	Percentage (%)
Retroelements	234,891	96,498,034	8.59
SINEs	2,311	291,064	0.03
LINEs	133,634	37,252,295	3.31
LTR elements	98,946	58,954,675	5.25
DNA transposons	7,445	1,092,740	0.10
Rolling-circles	1,858	1,015,043	0.09
Unclassified	89,799	46,879,085	4.17
Total interspersed repeats		144,469,859	12.85
Small RNA	749	82,339	0.01
Satellites	7,681	5,697,135	0.51
Simple repeats	235,850	13,986,714	1.24
Low complexity	49,091	3,115,412	0.28
Bases masked		168,298,524	14.97

### Gene model annotation

To generate a gene model annotation for the white-crowned sparrow assembly, various sources of evidence and different methodological approaches were integrated, and results consolidated to produce a non-redundant prediction. First, we performed an Iso-seq gene model annotation, following the nf-core/isoseq pipeline for Iso-seq data processing (https://github.com/nf-core/isoseq)^31^. In short, raw Iso-seq subreads were converted to CCS using default parameters and subsequently to FLNC reads. LIMA was then used to identify and remove barcodes and primer sequences. Given the library preparation kit used in our study, poly-A clean-up was run with primers suggested by TAMA toolkits^32^ for optimized retention of transcripts. The sequences were then mapped to the genome assembly using minimap2^33^, followed by processing with TAMA collapse and TAMA merge. Annotations that were created by subreads belonging to the same tissue were then merged, and annotations further merged across tissues.

Furthermore, we used the BRAKER (v2.1.6) annotation pipeline^34^ with ETP mode using transcriptomic evidence and protein homology evidence that was retrieved from closely-related reference species. The transcriptomic evidence was acquired from the three RNA-seq tissue samples that were mapped to the genome assembly using STAR (version 2.7.8a) with default parameters [12]. The large protein database includes OrthoDB vertebrate as well as chicken (GRCg6a) and zebra finch (bTaeGut1.4.pri). The aligned RNA-seq and protein database was used to support the training of GeneMark-ETP (version 4.71_lic)^35^, followed by AUGUSTUS (version 3.4.0) training and prediction with the same extrinsic information. Augustus training was run with “–species chicken” parameters. Using the BRAKER pipeline, an *ab initio* prediction was also generated^36^.

In addition, the transcript alignments were further utilized to detect splice junctions using portcullis (1.2.4). The results across multi-samples contributed to a unified set of annotation using PsiCLASS (v1.0.3)^37^. We then predicted open reading frames (ORF) using Transdecoder (5.5.0) (https://github.com/TransDecoder/TransDecoder) with an additional search for known proteins using Swiss-Prot (uniprot_sprot, retrived 2023 May) or pfam (3.1b2) using blastp (2.10.0+)^38^ or hmmscan (3.3.2)^39^. Gth (GenomeThreader 1.7.1) was also used to gain a protein alignment based gene structure prediction using the predicted protein sequences (https://genomethreader.org/).

Finally, the results of the above-mentioned predictions were all combined to a consensus annotation using EVM (EVidenceModeler-v2.0.0). We combined different sources of annotations, including the Iso-seq alignment, transcript alignment, protein alignment, GeneMark, and BRAKER predictions (both *ab initio* and with evidence). The BUSCO score for the transcriptome annotation using ‘*aves*’ database for assessment) shows 95.1% complete, 2.2% fragmented and 2.7% missing BUSCOs (Fig. 3). In total, the annotation resulted in 25,044 genes and 201,833 exons, with an average gene length of 19382.32 bp, an average exon count of 8.06 per gene, and an average exon length of 217.85 bp (Figure S3). The overall noncoding features of the annotation were predicted using CPC2 (0.1)^40^. CPC2 is a species-neutral approach to generate accurate assessment of the coding ability of RNA transcripts that were annotated by abovementioned sources in a fast manner. In total, we identified 18,674 coding genes and 6,370 noncoding genes. In addition, 495 tRNA were detected by using tRNAscan-SE and the details of 737 noncoding sequences (e.g. rRNA) were identified with the Rfam library using Infernal (Supplementary file 2)^41^. We show that overall distribution of gene features correlates with chromosome size (Fig. 4). In other words, the total number of genes is positively correlated with chromosome length, while the gene density is negatively correlated with chromosome length, with micro-chromosomes (e.g. 25, 27, 28, 29) exhibiting high density of gene features (Figure S4) as has been shown for chicken, turkey (*Meleagris gallopavo*) and barn swallow (*Hirundo rustica*)^42–44^.

## Data Records

The data presented in this paper were deposited in National Center for Biotechnology Information (NCBI) databases, with all sequences found under project accession number PRJNA889240. The Whole Genome Shotgun project has been deposited at GenBank under the accession JAPPSN000000000. The version described in this paper is version JAPPSN010000000, the GenBank sequence accession is GCA_028769735.1^45^. All sequence data used in the study is available under accession number SRP401897^46^, including the RNA-seq data under SRR21858074^47^, SRR21858075^48^ and SRR21858076^49^ the Iso-seq data under SRR21856897^50^, SRR21856898^51^, SRR21856899^52^ the whole genome sequencing data is available under SRR25788565^53^.

## Technical Validation

In order to assess the quality of the *Zonotrichia leucophrys* genome assembly, we used multiple methods and datasets for validation. Whole genome alignment to some closely related avian species was performed, including zebra finch (*Taeniopygia guttata*, bTaeGut1.4.pri, RefSeq accession: GCF_003957565.2)^54^, and white-throated sparrow (*Zonotrichia albicollis*, Zonotrichia_albicollis-1.0.1, Ensembl 108: GCA_000385455.1)^55^. NUCmer (NUCleotide MUMmer) aligner built in MUMmer (version 3.1)^56^ was used with default parameters. The percentage of total aligned bases to zebra finch and white-throated sparrow is 82.43% and 80.38%, respectively.

We then filtered the alignment for the minimum alignment identity at 30%. A DOT plot was used to visualize the cross-species alignment by adapting R code from dotPlotly (https://github.com/tpoorten/dotPlotly) with alignment cut off: queries with total alignments >80000 bp, minimum alignments >3000 bp.

To evaluate the quality of the RNA-seq data, FastQC (v0.11.7)^57^ and QualiMap (v.2.2.1)^58^ were used to assess the sequence and mapping quality, respectively. As shown in Figure S5, the input RNA-seq data has high quality, as demonstrated by the statistics of reads, e.g. base quality. The RNA-seq data was mapped to our assembled genome using STAR (version 2.7.8a)^24^. The input raw reads and mapping quality are summarized in Table 4, with an average uniquely mapping rate of 90.98%, indicating good quality and successful alignments to the genome assembly. Similarly, the short-read whole-genome sequencing data were mapped to the final assembly and then assessed for mapping quality. BWA-MEM^59^ was used for mapping with recommended parameters, and the percentage of mapped reads was 99.4% with a mean mapping score of 22.07.

**Table 4:** Validation of the white-crowned sparrow (*Zonotrichia leucophrys gambelii*) RNA-seq dataset.

Sample type	Number of input reads (pairs)	Uniquely mapped reads	Number of total splices	Mismatch rate per base
Gonad	33,585,925	92.25%	31,206,515	0.67%
Hypothalamus	34,035,354	89.73%	20,131,958	0.62%
Liver	34,085,391	90.97%	29,982,801	0.57%

### Supplementary information

Supplementary file 1

Supplementary file 2

## Data Availability

The majority of the data analyses were completed using standard bioinformatic tools running on the Linux system. The version and code/parameters of the main software tools are described in the text. Additional scripts used to generate the results and the figures can be found in the github repository: https://github.com/wzuhou/Genome_assembly_annotation. In addition, a diagrammatic pipeline is available on the home page (https://github.com/wzuhou/Genome_assembly_annotation/blob/main/README.md#pipeline).
