# Supplementary material for: A chromosome-level genome assembly of a free-living white-crowned sparrow (Zonotrichia leucophrys gambelii)
Source: Sci Data. 2024 Jan 18;11:86. doi: 10.1038/s41597-024-02929-6 (PMC10796373; doi:10.1038/s41597-024-02929-6)

**Supplementary file**

**Figure S1**. Interaction matrix shows the Omni-C results of White-Crowned Sparrow genome.

**Figure S2.** Alignment between Gambel’s White-Crowned Sparrow (GWCS) and zebra finch W and micro chromosomes.

**Figure S3**. Gene annotation features of White-crowned sparrow assembly.

**Figure S4**. Gene density across chromosomes

**Figure S5**. Report of quality control for RNA sequencing data.


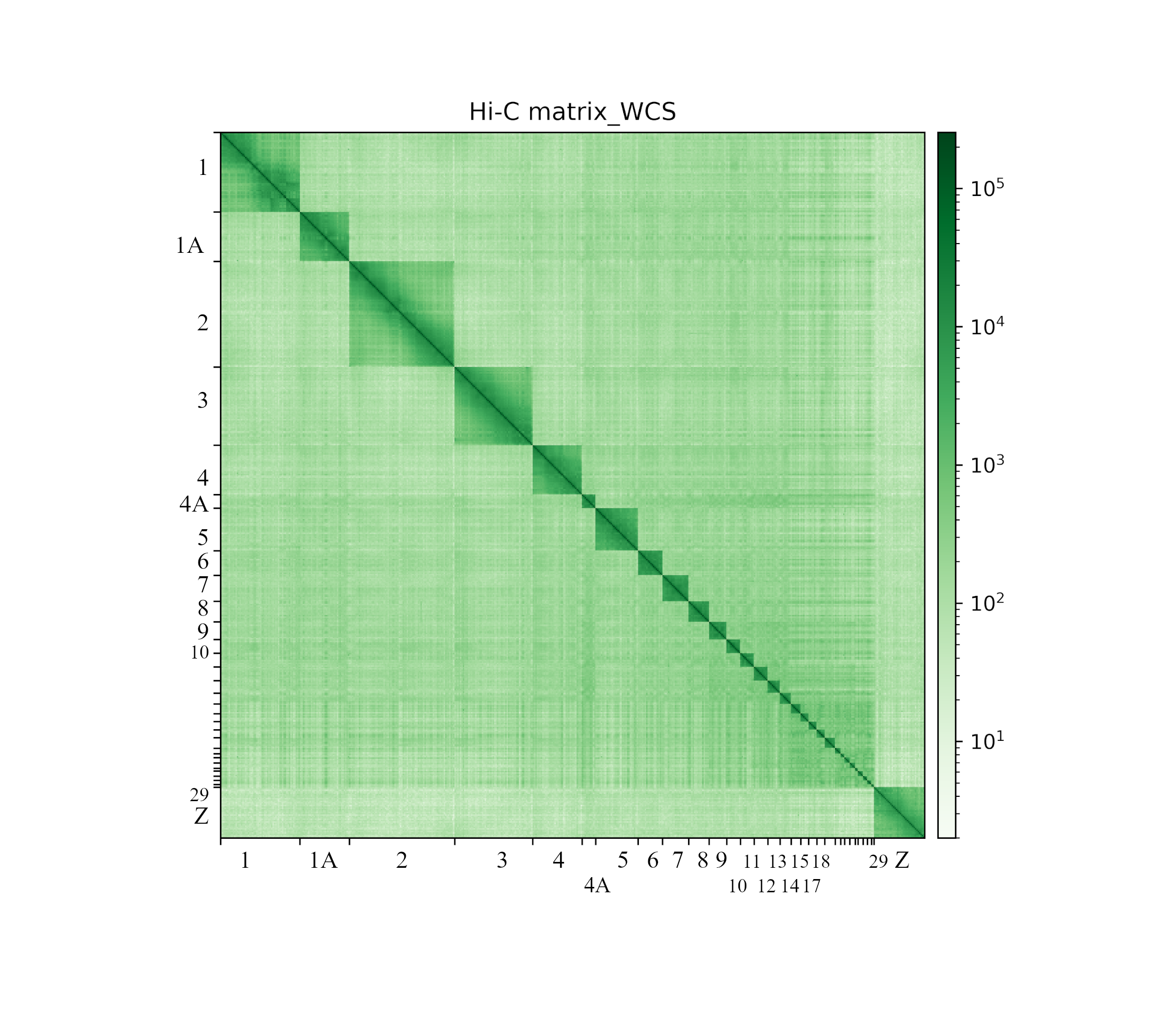
**Figure S1**. Interaction matrix shows the Omni-C results of White-Crowned Sparrow genome.

##
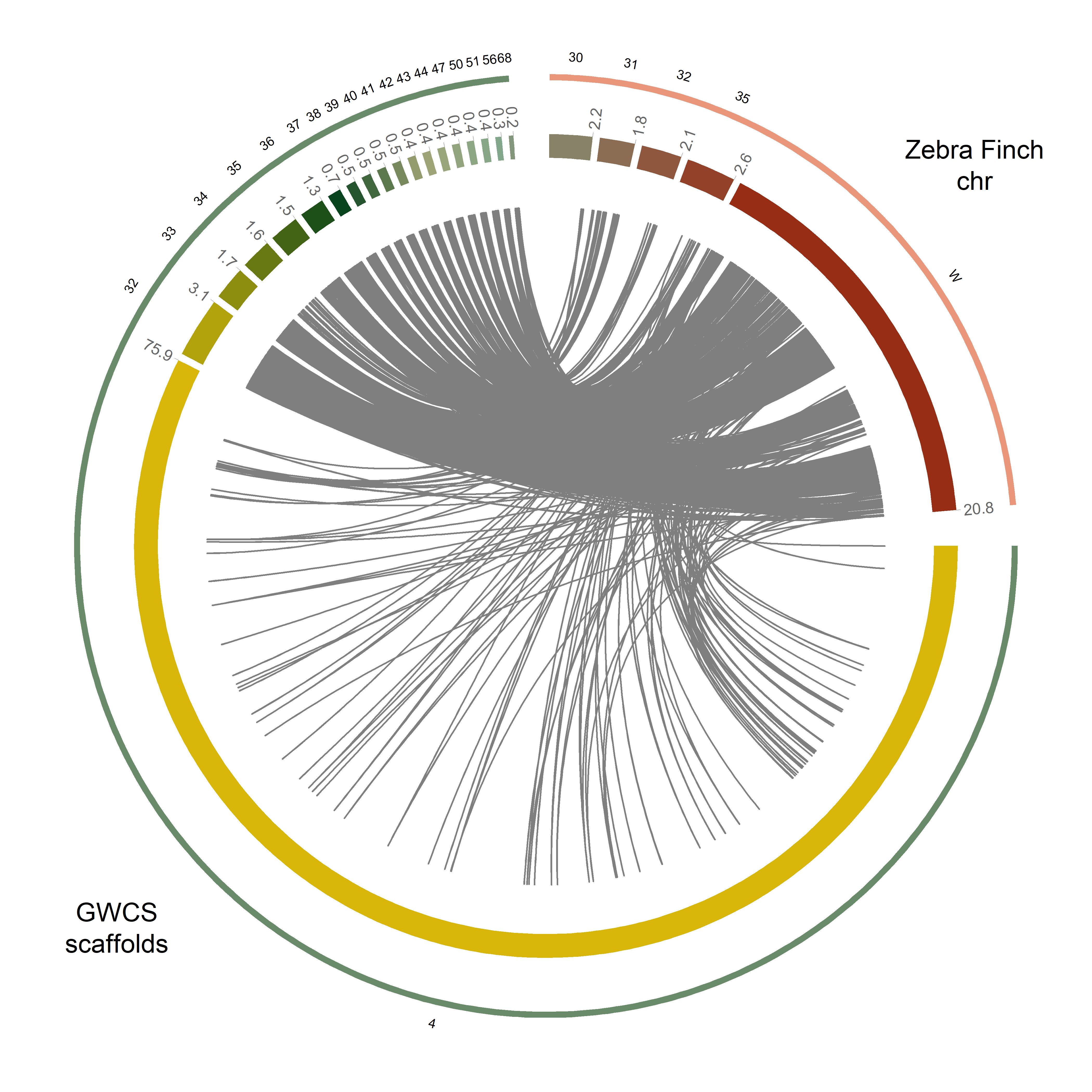
Figure S2. Alignment between Gambel’s White-Crowned Sparrow (GWCS) and zebra finch W and micro-chromosomes. Links show the alignment between scaffolds of GWCS (highlighted by outer green track) and zebra finch W chromosome, and micro-chromosomes 30, 31 32, 35 (highlighted by outer pink track). The length of each sequence is annotated in Mb. We filtered out alignments less than 1000 bp and queries with total alignments less than 80,000 bp. In addition, scaffold 4 of GWCS represents the Z chromosome, but due to the sequence homology there is excessive alignment to W chromosome.


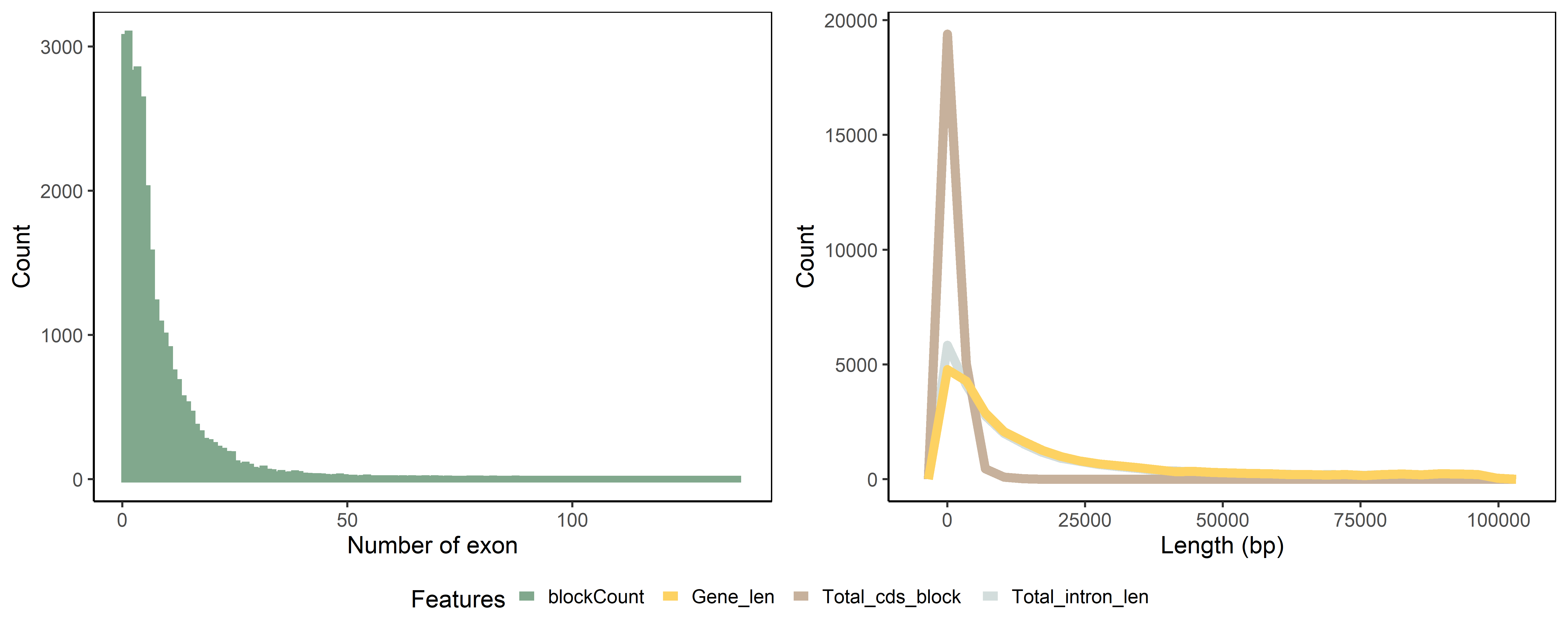


## Figure S3. Gene annotation features of the White-crowned sparrow assembly. The left figure shows the number of exons for each gene, and the right figure shows the length of different features in bp, including the length of gene (Gen_len), total length of cds (Total_cds_block), total length of intron (Total_intron_len).

##
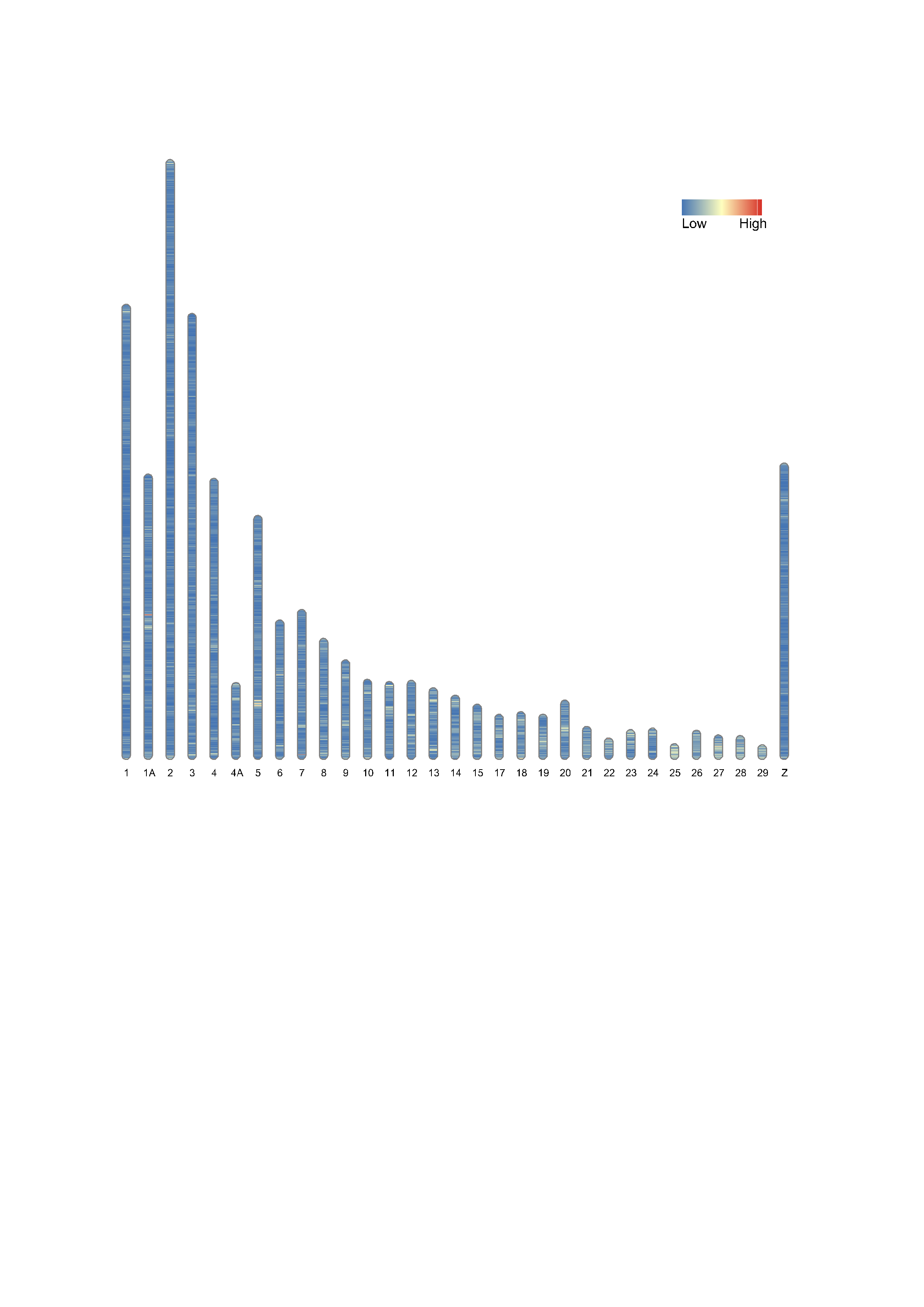
Figure S4. Gene density across the chromosomes. Only the prospective chromosomes are presented here.

## Figure S5. Report of quality control for RNA sequencing data.


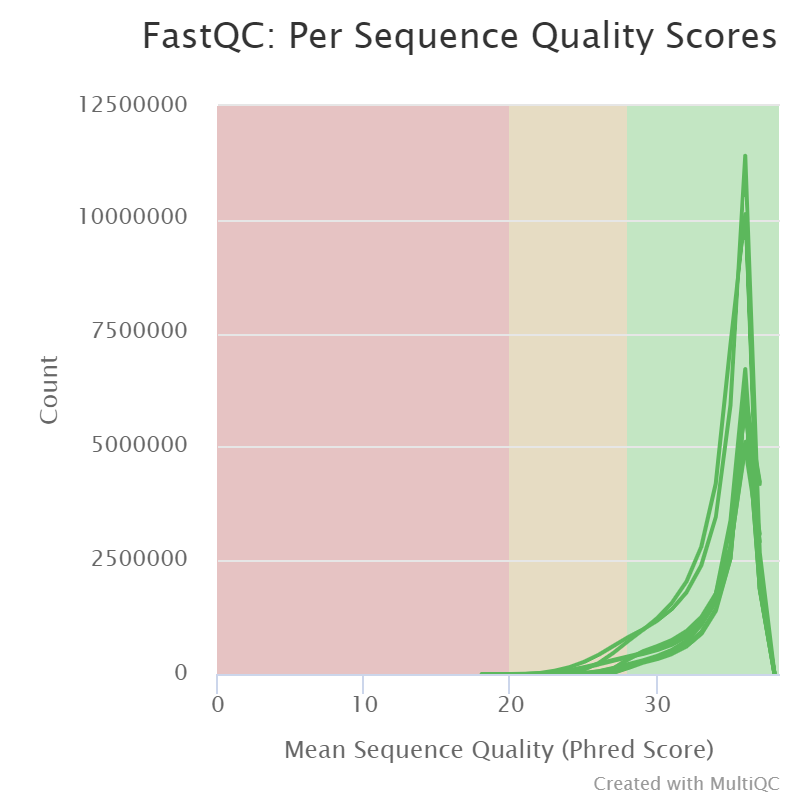

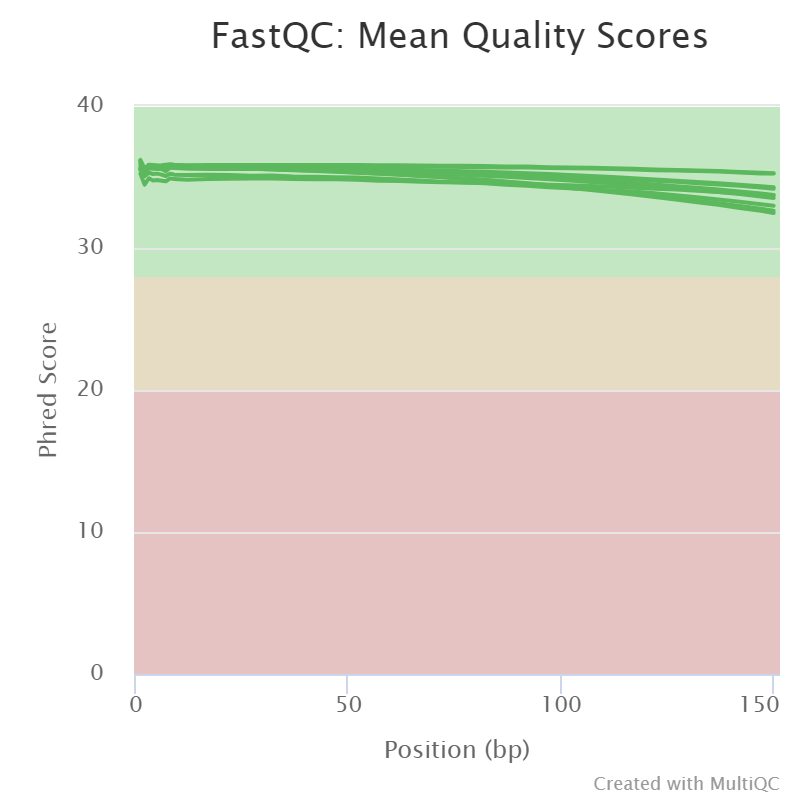

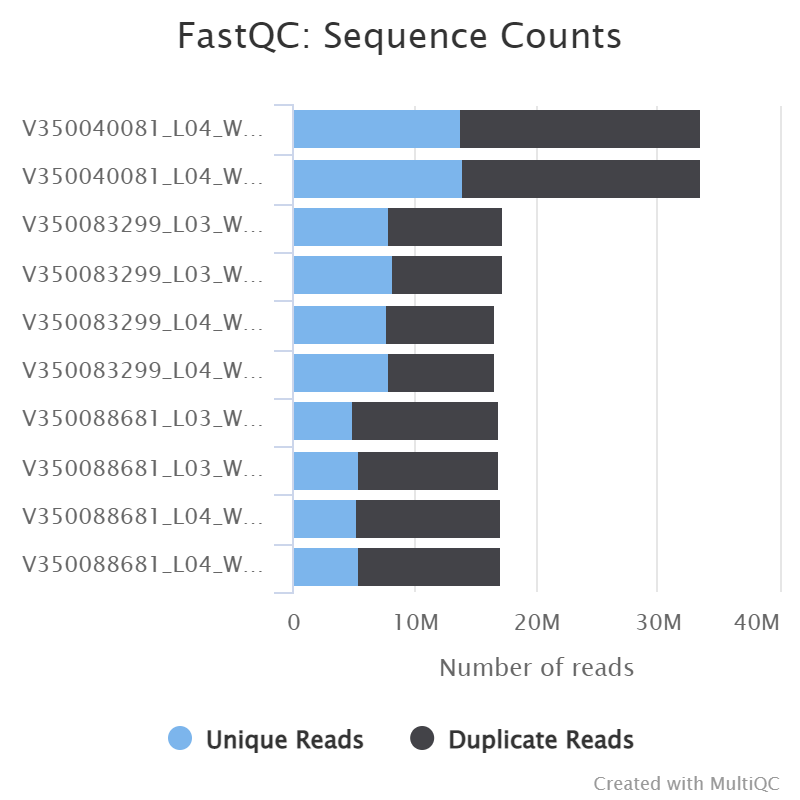

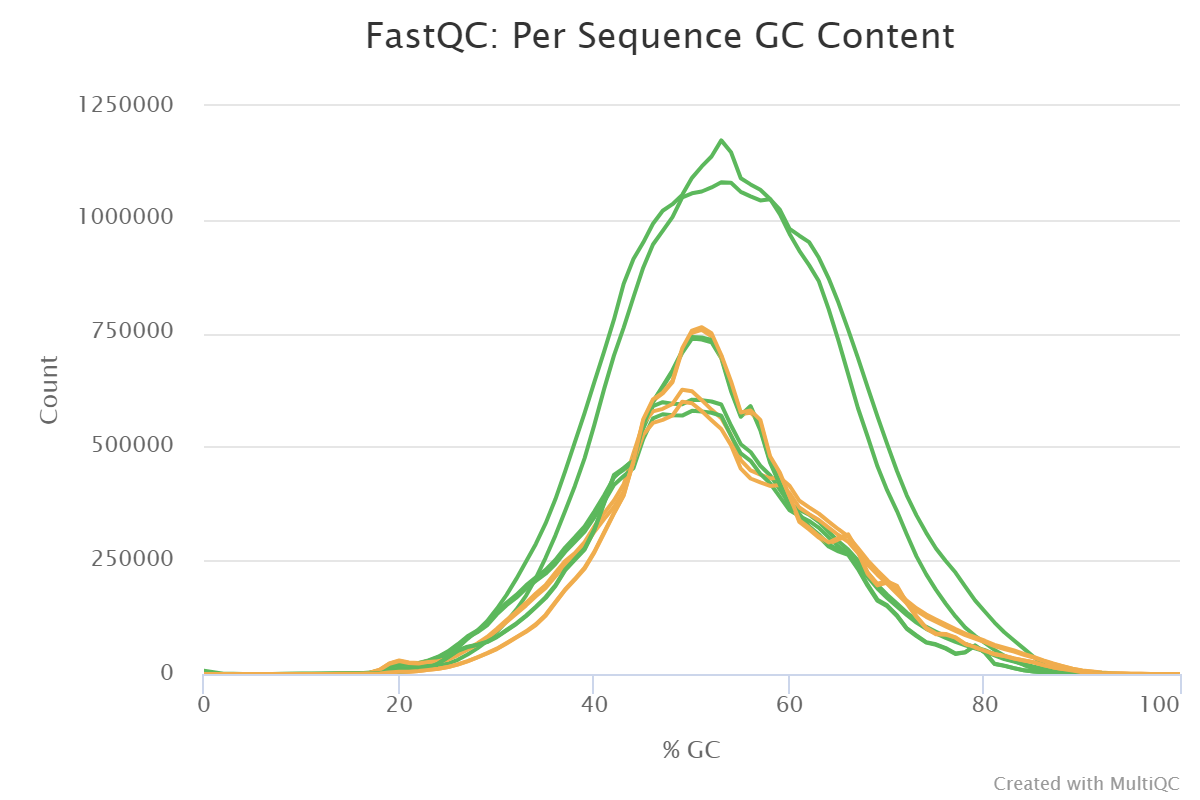

Supplement: Supplementary file 1 [file 41597_2024_2929_MOESM1_ESM.docx]
